# Fatigue in myasthenia gravis: risk factors and impact on quality of life

**DOI:** 10.1002/brb3.538

**Published:** 2016-08-02

**Authors:** Sarah Hoffmann, Johanna Ramm, Ulrike Grittner, Siegfried Kohler, Jana Siedler, Andreas Meisel

**Affiliations:** ^1^ NeuroCure Clinical Research CenterCharité – Universitätsmedizin BerlinBerlinGermany; ^2^ Department of NeurologyCharité – Universitätsmedizin BerlinBerlinGermany; ^3^ Center for Stroke Research (CSB)Charité – Universitätsmedizin BerlinBerlinGermany; ^4^ Department for Biostatistics and Clinical EpidemiologyCharité – Universitätsmedizin BerlinBerlinGermany

**Keywords:** activities of daily living, cohort studies, depression, fatigue, myasthenia gravis, quality of life

## Abstract

**Objectives:**

Emerging evidence suggests that fatigue in myasthenia gravis (MG) is a relevant problem that negatively impacts activities of daily living (ADL). The relationship between fatigue and quality of life (QoL) has never been systematically explored in MG patients. The study aimed to assess the prevalence of fatigue and its relation to ADL and QoL as well as to identify factors associated with fatigue in MG.

**Material and Methods:**

This was a cross‐sectional observational study in patients with confirmed diagnosis of MG independent of disease severity. Prevalence of fatigue was assessed using the Chalder Fatigue Scale (CFQ). Impact of fatigue on ADL and QoL was assessed by the MG activities of daily living profile (MG‐ADL) and the MG‐specific quality‐of‐life instrument (MG‐QoL), respectively. Association of fatigue with sociodemographics, clinical characteristics of MG, and comorbidities including mood and anxiety disorders as well as sleep disorders was investigated using multivariable logistic regression analyses.

**Results:**

Overall, 200 MG patients were included. The observed rate of fatigue was 56.1%, of those 70.4% fulfilled the criteria of chronic fatigue (CF) with a duration of ≥6 months. Relevant fatigue was strongly associated to ADL and QoL. Factors associated with relevant fatigue were disease severity and depressive state. Furthermore, positive muscle‐specific tyrosine kinase (MuSK) antibody status showed a strong association with relevant fatigue.

**Conclusions:**

MG patients have a high prevalence of fatigue which negatively impacts ADL and QoL. MG‐specific clinical characteristics are related to fatigue and might help to identify MG patients at risk for fatigue.

## 1 Introduction

Fluctuating, painless muscle weakness is usually referred to as cardinal symptom of myasthenia gravis (MG; Cejvanovic & Vissing, 2014; Grob, Brunner, Namba, & Pagala, 2008). However, the clinical picture of MG is more complex and emerging evidence recognizes fatigue as a relevant problem in MG (Elsais, Wyller, Loge, & Kerty, 2013; Paul, Cohen, Goldstein, & Gilchrist, 2000). Previous studies reported fatigue prevalence rates between 75% and 89% in MG patients (Kluger, Krupp, & Enoka, 2013) and qualitative data suggest that, in some patients, fatigue has a greater impact on daily living of MG patients than has muscle weakness (Barnett, Bril, Kapral, Kulkarni, & Davis, 2014; Zwarts, Bleijenberg, & van Engelen, 2008). However, overall literature on fatigue in MG is scarce and its impact on activities of daily living (ADL) and quality of life (QoL) has never been systematically explored. This might be due to various reasons. Fatigue is a complex, nonspecific, and highly subjective symptom and therefore difficult to evaluate and quantify (Krupp, LaRocca, Muir‐Nash, & Steinberg, 1989; Norheim, Jonsson, & Omdal, 2011). The fluctuating and effort‐dependent nature of fatigue makes it even more difficult to separate fatigue from muscle fatigability in MG. We follow recent proposals to use the term fatigue to refer to subjective sensations of exhaustion and muscle fatigability to refer to objective changes in muscle performance (Kluger et al., 2013). Other related phenomena such as depression and sleep disorders need to be distinguished from fatigue and should therefore be included as covariates when assessing fatigue (Kluger et al., 2013). Finally, the understanding of the pathophysiology of fatigue is limited. MG is an autoimmune‐mediated disease with autoantibodies directed against components of the postsynaptic muscular endplate (Szczudlik et al., 2014). The most likely confinement to the peripheral nervous system makes hypotheses on the pathophysiology of fatigue in MG particularly challenging.

The aim of the present study was to assess the prevalence of fatigue and its impact on ADL and QoL in a large cohort of MG patients as well as to identify factors associated with fatigue including MG‐specific clinical characteristics as well as potential confounders such as mood and sleep disturbances (Elsais et al., 2013; Kluger et al., 2013).

## 2 Material and Methods

### 2.1 Patients

This was a cross‐sectional, observational study performed at the certified Integrated Center for Myasthenia gravis (IMZ) of the Charité – Universitätsmedizin Berlin, Germany. Patients over the age of 18 years with confirmed diagnosis of myasthenia gravis were included independent of disease duration and severity (excluding myasthenic crisis). Patients were consecutively screened at the IMZ clinic between December 2012 and December 2013.

Sociodemographics (age, sex), current MG‐specific medication (cholinesterase inhibitors, glucocorticoids, and long‐term immunosuppressants), current comedication (antidepressants, NSAIDs, and opioids), and comorbidities (other immunopathies, cardiac insufficiency, and malignancies) were collected in a database. Clinical assessment was performed using the MGFA classification for disease classification (Jaretzki et al., 2000) and the QMG score for disease severity. Using the MGFA classification, patients were grouped into ocular (MGFA I) or generalized MG patients (MGFA II–IV) at the time of study inclusion. According to current recommendations, we used the MGFA classification by employing the most severely affected muscles to define the patient's MGFA class (the “maximum severity” designation being made historically; Jaretzki et al., 2000). Thereby, patients were only assigned to MGFA I if symptomatology was restricted to purely ocular symptoms throughout the patient's entire disease history (ocular MG). Correspondingly, patients with generalized symptoms (affecting limb/axial and/or bulbar/respiratory muscles) throughout their disease history were assigned to generalized MG. An exception to this approach was made in patients without any detectable myasthenic weakness under current medication who were assigned to “pharmacological remission.”

### 2.2 Questionnaires

All patients completed the self‐assessment questionnaires at the IMZ. Completion of questionnaires required approximately 30 min. If available, validated German versions of questionnaires were used, as in the case of the Chalder Fatigue Scale (CFQ; Martin, Gaab, Rief, & Brähler, 2010) and the Hospital Anxiety and Depression Scale (HADS; Hinz & Schwarz, 2001). The other questionnaires (Insomnia Severity Index, MG activities of daily living profile, and MG‐specific quality‐of‐life instrument) were translated using forward–backward translation procedure by two physicians (SH, JR) who are both native German with excellent knowledge of English. The translations were reviewed by the principal investigator (AM). In case of discrepancies, a reconciled version was produced by a consensus conference.

#### 2.2.1 Chalder Fatigue Scale

For assessing fatigue, the CFQ presented by Chalder et al. (1993) was used. The CFQ is a self‐administered questionnaire for measuring the severity of physical (CFQ‐P) and mental (CFQ‐M) fatigue within both clinical and epidemiological populations (Jackson, 2015). It consists of 11 items that are answered on a 4‐point scale ranging from the asymptomatic to maximum symptomatology, such as “less than usual,” “no more than usual,” “more than usual,” and “much more than usual” offering the option of binary scoring (0 0 1 1) or Likert scoring (0 1 2 3). We used the binary scoring (total score 0–11) to define caseness in patients with a score of 4 points or higher as proposed by Chalder et al. and the Likert scoring (total score 0–33) to evaluate mean fatigue scores in the different fatigue domains. To differentiate between transient and chronic fatigue (CF), we assessed fatigue duration by asking if symptoms were present for <6 months or longer.

#### 2.2.2 MG‐specific quality‐of‐life instrument

MG‐specific quality‐of‐life instrument (MG‐QoL) is a 15‐item questionnaire encompassing physical and psychological domains of MG to assess disease‐specific QoL in MG patients (Burns, Conaway, Cutter, & Sanders, 2008). Rating consists of a 5‐point scale ranging from 0 (“not at all”) to 4 (“very much”) as to the degree to which patients agree with the given statement summing up to a total score of 0–60 points.

#### 2.2.3 MG activities of daily living profile

Impact on daily living was assessed using the MG activities of daily living profile (MG‐ADL; Wolfe et al., 1999). It is an eight‐question survey of symptom severity, with each response graded from 0 (normal) to 3 (most severe). Questions include ocular, oropharyngeal, respiratory, and extremity functions. Total MG‐ADL score ranges from 0 to 24.

#### 2.2.4 Hospital anxiety and depression scale

Hospital anxiety and depression scale is a self‐assessment mood scale developed to identify cases of anxiety disorders and depression among patients in nonpsychiatric hospital clinics (Zigmond & Snaith, 1983). It is divided into an anxiety subscale (HADS‐A) and a depression subscale (HADS‐D) both containing seven items (Bjelland, Dahl, Haug, & Neckelmann, 2002) with a 0‐ to 3‐point Likert response format summing up to a total score of 21 for each subscale. A cut‐off score of 8 or higher on each subscale was used to define caseness of anxiety and depression, respectively (Bjelland et al., 2002). Symptoms of mood disorders likely to be present in somatic disorders (e.g., insomnia, fatigue, anorexia) are excluded in the HADS.

#### 2.2.5 Insomnia Severity Index

Sleep disturbances were assessed using the ISI. It is a seven‐item self‐report instrument targeting the subjective symptoms and consequences of insomnia (Bastien, Vallieres, & Morin, 2001). Using the Likert scale, each item is rated between 0 to 4 points summing up to a total score range from 0 to 28. We used a cut‐off score of 10 points to define caseness of sleep disturbances (Morin, Belleville, Belanger, & Ivers, 2011).

### 2.3 Statistical analysis

**Figure 1:**
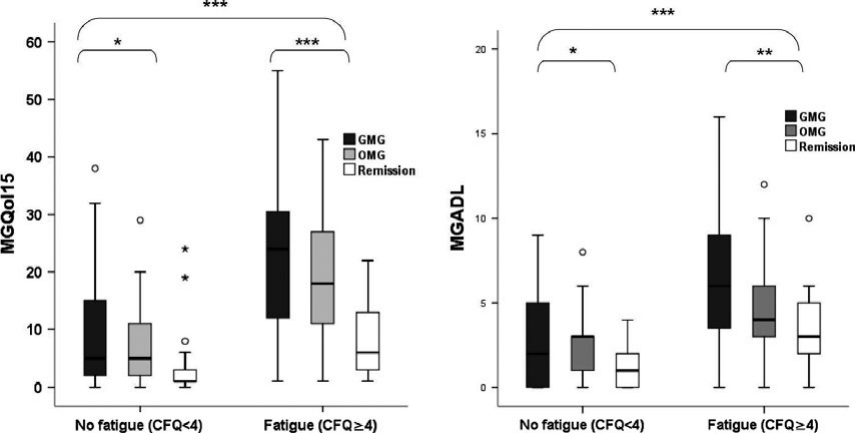
Subgroup analyses of MGQoL15 and MGADL stratified by relevant fatigue and disease classification. MGQoL15 and MGADL scores are significantly higher in patients with relevant fatigue. Within the group of patients with relevant fatigue, patients with GMG have significantly higher MG‐QoL and MGADL scores compared to patients with in pharmacological remission. *p ≤ .05, **p ≤ .01, ***p ≤ .001. GMG, generalized myasthenia gravis; OMG, ocular myasthenia gravis; CFQ, Chalder Fatigue Scale; MGQoL15, MG‐specific quality‐of‐life instrument; MGADL, MG activities of daily living profile

Descriptive analyses included reports of means and standard deviation, median and interquartile range (IQR), or absolute and relative frequencies depending on the scale and distribution of variables. We compared sociodemographic characteristics, questionnaire scores, and clinical characteristics between the myasthenia subgroups using Fisher's exact test, chi‐square test, one‐way ANOVA, or Kruskal–Wallis test where indicated (Tables 1 and 2). To analyze association of characteristics with clinical‐relevant fatigue, we used *t*‐test, Mann–Whitney test, Fisher's exact test, or chi‐square test (Table 3). In multiple logistic regression analysis we included and tested variables that were associated with relevant fatigue in univariate analysis (*p *< .05). As all six MuSK‐ab‐positive patients had relevant fatigue, the problem of quasi‐separation appears resulting in nonexistence of OR and CI estimates. Therefore, Firth logistic regression was used (Stata/IC 13.1 command firthlogit), estimating ORs and CIs with penalized likelihood estimation methods (Heinze & Schemper, 2002). Only significant variables remained in the final model with the exception of MuSK‐ab which was forced into the model. We used a two‐sided significance level of alpha = 0.05. Linear regression was used to analyze association of relevant fatigue with ADL and QoL adjusted for MG type (one regression model for each outcome, Fig. 1). No adjustment for multiple testing was applied as this is an exploratory study. Statistical tests were performed using SPSS 22 (IBM, Armonk, NY, USA).

**Table 1:** Baseline characteristics

Characteristic	All	GMG	OMG	Remission	*p*‐value
*n* (%)	200	119	39	42	
Age, year, mean (*SD*)	58 (17)	56 (17)	60 (15)	61 (17)	.235
Age at disease onset, mean (*SD*)	48 (20)	45 (21)	53 (17)	52 (20)	.032
Female sex, *n* (%)	107 (53.5)	76 (63.9)	12 (30.8)	19 (45.2)	.001
Disease duration, year, median (IQR)	6 (2–15)	7 (3–16)	3 (2–10)	6 (2–10)	.014
Antibody status, *n* (%) (1 missing)					
AchR	166 (83.4)	98 (83.1)	30 (76.9)	38 (90.5)	.258
MuSK	6 (3.0)	5 (4.2)	1 (2.6)	—	.535
Negative	27 (13.6)	15 (12.7)	8 (20.5)	4 (9.5)	.322
Medication, *n* (%)					
Cholinesterase inhibitors	173 (86.5)	107 (89.9)	37 (94.9)	29 (69.0)	.001
Glucocorticoids	111 (55.5)	68 (57.1)	23 (59.0)	20 (47.6)	.502
Immunosuppressants	116 (58.0)	81 (68.1)	10 (25.6)	25 (59.5)	<.001
None	4 (2.0)	1 (0.8)	–	3 (7.1)	.064
Thymectomy, *n* (%) (9 missings)	99 (51.8)	68 (59.6)	9 (24.3)	22 (55.0)	.001
Thymoma	24 (24.2)	18 (75.0)	3 (12.5)	3 (12.5)	.380
Thymus hyperplasia	31 (31.3)	21 (67.7)	2 (6.5)	8 (25.8)	.736
Comedication, *n* (%)					
Antidepressants	18 (9.0)	15 (12.6)	2 (5.1)	1 (2.4)	.089
NSAIDs	13 (6.5)	8 (6.7)	2 (5.1)	3 (7.1)	.924
Opioids	8 (4.0)	7 (5.9)	1 (2.6)	0 (0.0)	.217

GMG, generalized myasthenia gravis; OMG, ocular myasthenia gravis; AchR, acetylcholine receptor; MuSK, muscle‐specific tyrosine kinase.

*p*‐values refer to overall group comparisons of patients with generalized MG, ocular MG, and pharmacological remission.

**Table 2:** Prevalence and severity of relevant fatigue, prevalence of mood, anxiety, and sleep disorders

Parameter	All	GMG	OMG	Remission	*p*‐value
*n*	196	116	39	41	
CFQ ≥ 4 items, *n* (%)	110 (56.1)	83 (71.6)	14 (35.9)	13 (31.7)	<.001
CFQ‐T score, mean (*SD*)	15.6 (6.2)	17.5 (6.0)	14.0 (5.6)	11.7 (5.0)	<.001
CFQ‐P score, mean (*SD*)	10.7 (4.5)	12.1 (4.3)	9.4 (4.1)	7.8 (3.7)	<.001
CFQ‐M score, mean (*SD*)	4.9 (2.3)	5.4 (2.4)	4.7 (2.2)	3.9 (1.9)	.002
CF ≥ 6 months within *n *= 108 patients with relevant fatigue, *n* (%) (2 missings)	76 (70.4)	59 (72.0)	9 (64.3)	8 (66.7)	.808
HADS‐D ≥ 8 points, *n* (%) (6 missings)	38 (19.6)	28 (24.6)	6 (15.4)	4 (9.8)	.093
HADS‐A ≥ 8 points, *n* (%) (6 missings)	54 (27.8)	41 (36.0)	7 (17.9)	6 (14.6)	.010
ISI ≥ 10 points, *n* (%)	83 (41.5)	61 (51.3)	12 (30.8)	10 (23,8)	.003

GMG, generalized myasthenia gravis; OMG, ocular myasthenia gravis; CFQ, Chalder Fatigue Scale; CFQ‐T, CFQ‐Total; CFQ‐P, CFQ‐Physical; CFQ‐M, CFQ‐Mental; CF, chronic fatigue (≥6 months); ISI, Insomnia Severity Index; HADS, Hospital Anxiety and Depression Scale; HADS‐D, HADS‐Depression; HADS‐A, HADS‐Anxiety; MG‐ADL, MG activities of daily living profile.

*p*‐values refer to overall group comparisons of patients with generalized MG, ocular MG, and pharmacological remission.

**Table 3:** Factors associated with relevant fatigue: univariate analysis

Characteristic	Relevant fatigue (CFQ ≥ 4)	No fatigue (CFQ < 4)	*p*‐value
*n* (%)	110 (56.1)	86 (43.9)	
Age, year, mean (*SD*)	59 (17)	58 (16)	.951
Age at disease onset, mean (*SD*)	48 (20)	48 (21)	.975
Female sex, *n* (%)	68 (66.0)	35 (34.0)	.003
Disease duration, year, median (IQR)	7 (3–14)	5 (2–15)	.881
QMG score, mean (*SD*)	9.9 (6.0)	4.4 (4.0)	<.001
ISI score, mean (*SD*)	11.6 (7.2)	5.5 (5.1)	<.001
ISI ≥ 10 points, *n* (%)	63 (79.7)	16 (20.3)	
HADS‐D, mean (*SD*)	6.5 (3.6)	2.2 (2.4)	<.001
HADS‐D ≥ 8 points, *n* (%)	34 (89.5)	4 (10.5)	
HADS‐A, mean (*SD*)	6.9 (3.8)	3.8 (3.2)	<.001
HADS‐A ≥ 8 points, *n* (%)	42 (77.8)	12 (22.2)	
Antibody status, *n* (%)			
AchR	85 (52.5)	77 (47.5)	.033
MuSK	6 (100)	0 (0.0)	.035
Negative	18 (66.7)	9 (33.3)	.225
Medication, *n* (%)			
Cholinesterase inhibitors	100 (59.2)	69 (40.8)	.031
Glucocorticoids	67 (60.4)	44 (39.6)	.172
Immunosuppressants	69 (61.1)	44 (38.9)	.104
None	1 (25.0)	3 (75.0)	.321
Thymectomy, *n* (%) (9 missings)	60 (63.2)	35 (36.8)	.050
Thymoma	11 (47.8)	12 (52.2)	.080
Thymus hyperplasia	18 (62.1)	11 (37.9)	.884
Comorbidities			
Other immunopathies	34 (68.0)	16 (32.0)	.050
Malignancies	3 (42.9)	4 (57.1)	.701
NYHA	5 (83.3)	1 (16.7)	.233
Comedication, *n* (%)			
Antidepressants	13 (72.2)	5 (27.8)	.149
NSAIDs	9 (75.0)	3 (25.0)	.174
Opioids	8 (100.0)	0 (0.0)	.011

QMG, quantitative myasthenia gravis score; ISI, Insomnia Severity Index; HADS, Hospital Anxiety and Depression Scale; HADS‐D, HADS‐Depression; HADS‐A, HADS‐Anxiety; AchR, acetylcholine receptor; MuSK, muscle‐specific tyrosine kinase; CFQ, Chalder Fatigue Scale.

### 2.4 Ethics

The study was approved by the ethics committee of the Charité – Universitätsmedizin Berlin (EA1/281/10). All patients gave written informed consent in accordance with the Declaration of Helsinki in its currently applicable form.

## 3 Results

### 3.1 Patient's demographic and clinical characteristics

Overall, 200 patients were included. Mean age was 58 years (*SD*: 17), 107 (53.5%) were female. Most patients were positive for AchR‐ab (*n *= 166, 83.4%), 6 (3.0%) patients were positive for MuSK‐ab, and 27 (13.6%) patients were double seronegative. About half of the patients (*n *= 99, 49.5%) had undergone thymectomy. Of those who had undergone thymectomy, 24 (24.2%) had thymoma and 31 (31.3%) patients revealed thymus hyperplasia in the histological examination. Among the patients taking long‐term immunosuppressant drugs (*n *= 116, 58%), 85 (42.5%) patients took azathioprine, 16 (8%) patients took methotrexate, 14 (7%) patients took mycophenolate mofetil, and 1 (0.5%) patient took cyclosporine A. Median disease duration was 6 years (IQR: 2–15). Table 1 shows the baseline characteristics for MG patients in total and stratified by GMG, OMG, and pharmacological remission.

### 3.2 Fatigue prevalence and severity

Relevant fatigue (CFQ ≥ 4 points on the binary scale) was present in 110 (56.1%) of MG patients (Table 2). Rates of relevant fatigue ranged from 31.7% in patients in pharmacological remission to 71.6% in patients with generalized MG (*p* < .001). GMG patients had significantly higher CFQ scores (Likert scale) in the physical as well as in the mental domain compared to remitted MG as well as OMG patients (Table 2). Within the group of patients with relevant fatigue, chronic fatigue (≥6 months) was similarly distributed among patients with GMG (72.0%) compared to patients with OMG (64.3%) or patients in pharmacological remission (66.7%).

### 3.3 Prevalence of mood, anxiety, and sleep disorders

Using a cut‐off score of ≥8 points on the HADS, rates for depression and anxiety in the total sample are 19.6% and 27.8%, respectively (Table 2). Rate of sleeping disturbances defined as a cut‐off score of ≥10 points on the ISI were 41.5%. Both, mood and sleeping disorders were more frequent in patients with GMG compared to those with OMG or in pharmacological remission.

### 3.4 Factors associated with relevant fatigue: univariate and multivariate analysis

The following characteristics were associated with relevant fatigue in univariate analysis (Table 3): female sex, GMG, positive AchR or MuSK antibody status, medication with cholinesterase inhibitors or opioids, thymectomy, presence of other immunopathies, higher QMG score, higher HADS‐D and HADS‐A scores, and higher ISI score. Multivariable logistic regression showed that higher QMG score and higher HADS‐D score were significantly associated with relevant fatigue after adjustment for each other and for positive MuSK‐ab status (Table 4). Positive MuSK‐ab status showed a strong association with relevant fatigue, although due to the low case number this finding did not reach statistical significance.

**Table 4:** Factors associated with clinically relevant fatigue: final multivariable analyses

Characteristic	Relevant fatigue (CFQ ≥ 4)OR (95% CI)
QMG score	1.17 (1.07–1.27)
HADS‐D	1.55 (1.31–1.82)
MuSK	15.63 (0.67–362.46)

QMG, quantitative myasthenia gravis score; HADS‐D, Hospital Anxiety and Depression Scale‐Depression; MuSK, muscle‐specific tyrosine kinase; CFQ, Chalder Fatigue Scale.

Firth regression model (*n *= 173), odds ratios (OR), and 95% CI (Tjur's *r* ^2^ = .45) (MuSK‐ab was forced into the model, otherwise only significant coefficients remained in the final model).

### 3.5 Fatigue and its association with quality of life and activities of daily living

Figure 1 shows that quality of life as measured by the MG‐QoL15 and activities of daily living as measured by the MG‐ADL scores were significantly higher (higher scores indicating more severe symptoms) in patients with relevant fatigue (*p* < .001). Within the group of patients with relevant fatigue, mean MG‐QoL score was 22.7 points in GMG patients compared to patients with OMG (19.3) and patients in pharmacological remission (8.6). Mean MG‐ADL score within the group of patients with relevant fatigue was 6.2 points in GMG patients compared to patients with OMG (4.8 points) and patients in pharmacological remission (3.3 points).

## 4 Discussion

Our study adds to the scarce literature on fatigue in MG by demonstrating a high prevalence of fatigue among MG patients and showing a negative impact of fatigue on QoL in MG patients. Furthermore, we identified disease severity and depressive state as independent risk factors associated with fatigue in MG patients.

The prevalence of fatigue in our MG cohort was 56.1%. A recent study by Elsais et al. (2013) using the same questionnaire in a Norwegian MG population reported a fatigue rate of 44%. The higher fatigue rate in our cohort might be explained by a higher disease severity. Elsais et al. (2013) only included patients with MGFA grade II or better, whereas we only excluded patients with myasthenic crisis. Consistently, a higher QMG score was associated with a higher prevalence of relevant fatigue. It is noteworthy, that the QMG score is an instrument to measure muscle fatigability, not fatigue. The authors are aware of the challenge of separating fatigue from muscle fatigability. Fatigue is a multidimensional concept covering both physical and psychological aspects making it difficult to assess fatigue in neurological diseases in general and in MG in particular. However, the high prevalence of fatigue in our cohort and its negative impact on ADL and QoL underline the need of further research on fatigue in MG. Moreover, one third of MG patients in pharmacological remission in our cohort suffered from relevant fatigue. Our finding is consistent with the results of Elsais et al. (2013) suggesting that factors other than myasthenic muscle weakness are involved in the pathogenesis of fatigue in MG. Furthermore, the distinction between fatigue and muscle fatigability might be of practical relevance, for example, might require different therapeutical approaches. To the best of our knowledge, there is no interventional study on fatigue in MG. One study reported an improvement of fatigue after supplementation of vitamin D3 (Askmark, Haggard, Nygren, & Punga, 2012). However, in this study fatigue was assessed using the MG composite scale, thereby mainly measuring muscle fatigability rather than fatigue. Awareness for the need of separating fatigue from muscle fatigability in MG is only beginning to rise. More studies on this topic are warranted to reproduce our findings or to unravel other factors associated with fatigue. If, for example, disease severity showed a consistent association with fatigue in MG, an escalation of symptomatic and/or immunosuppressive therapy could be an approach to treat fatigue in MG. Treatment of fatigue itself should consist of a stepwise approach of psychological, physical, and pharmacological interventions. The latter is particularly challenging in MG because MG symptoms can be worsened by a variety of drugs.

We included measures of mood and sleep disturbances as covariates in our analyses as recommended by other research groups (Elsais et al., 2013; Kluger et al., 2013). Depressive state was associated with higher prevalence of fatigue. The association between fatigue and depression is widely accepted and mainly attributed to the overlap in symptomatology between fatigue and depression (such as physical fatigue; Norheim et al., 2011). Rate of depressive state was 19.6% in our MG cohort and thereby comparable to those in the general German population (Hinz & Brahler, 2011). It has long been suggested that depression is common among MG patients (Chafetz, 1966; Meyer, 1966). However, more recent studies show inconsistent results. Although some studies report markedly higher depression rates of up to 33% in MG patients (Fisher, Parkinson, & Kothari, 2003; Magni et al., 1988), others have found no increased frequency of depression in MG patients over the general population (Doering, Henze, & Schussler, 1993; Tennant, Wilby, & Nicholson, 1986). These conflicting results might be partially attributable to the use of different measures of depression across the studies. The use of the HADS which was developed to identify caseness of anxiety disorders and depression among patients in nonpsychiatric hospital clinics and therefore excludes physical symptoms might be particularly suitable to overcome the overlap in symptomatology between MG, fatigue, depression, and sleep disorders.

Positive MuSK antibody status shows a strong influence on relevant fatigue, although this finding did not reach statistical significance. This might be due to the low case number (*n *= 6). However, the prevalence of MuSK‐positive patients among AchR‐negative patients was 17.6% and thereby comparable to the prevalence of other European MG cohorts (Kostera‐Pruszczyk et al., 2008; Niks, Kuks, & Verschuuren, 2007). Attempts have been made to better characterize MG patients by their antibody status and differences were found for patterns of involved muscles, disease severity, as well as treatment responses (Ohta et al., 2007). Interestingly, all of the MuSK‐positive MG patients reported relevant fatigue. Due to the low case number, this finding needs to be interpreted with caution but MuSK‐ab and their association with relevant fatigue seem to be a promising target for further investigations. Future studies on fatigue in MG should furthermore consider newly described antibodies such as anti‐LRP4 and anti‐Agrin antibodies (Gasperi et al., 2014).

Our study has several limitations that need to be acknowledged. The study design did not include a healthy control group to compare our data with. However, the herein used CFS allows for comparisons of fatigue prevalence in the general population (Martin et al., 2010) and other MG cohorts (Elsais et al., 2013). Main objectives of our study were furthermore the identification of clinical factors associated with fatigue in MG assessing QoL and ADL with MG‐specific self‐questionnaires which could not have been performed in a group of healthy controls. Patients were grouped into OMG and GMG patients by employing the most severely affected muscles to define the patient's MGFA class. This might have introduced a bias as GMG patients could have presented with purely ocular symptoms at study inclusion. However, in our experience, a mild affection of other than purely ocular muscles is noticeable for most GMG patients. For some of the questionnaires used in this study (ISI, MG‐ADL, MG‐QoL), validated German versions were not available which might have influenced the results. Statistical analysis did not include multiple testing. However, this was an exploratory analysis without any predefined hypotheses and predictors for relevant fatigue were identified using multivariable regression analysis. Further limitations include potential shortcomings concerning the clinical factors included in our analyses. For example, comedication did not consider hypnotics and betablockers despite their reported negative effect on fatigue (Braley, Segal, & Chervin, 2015; Tang, Yu, & Yeh, 2010). The test battery with five self‐assessment questionnaires might have influenced the results. Patients were free to decide on the order of questionnaire completion, therefore, we cannot entirely exclude an overestimation of mental fatigue due to prior concentration on completion of other questionnaires. However, increasing emphasis has been placed on self‐reporting instruments as they allow for an assessment of highly subjective conditions such as fatigue as well as for an evaluation of the impact of diseases such as QoL and ADL (Wolfe et al., 1999).

In conclusion, our study emphasizes the importance of fatigue in MG even in patients in pharmacological remission and its impact on ADL and QoL. MG‐specific clinical characteristics seem to have an influence on fatigue and might help to identify MG patients at risk for fatigue. Fatigue could be misinterpreted as muscle fatigability or mood disorders. Therefore, depressive state should be assessed excluding physical symptoms of depression in order to avoid overestimation of depressive state in MG patients. Future clinical trials in MG should include fatigue as endpoint in order to assess if fatigue changes with improvement of disease severity or whether fatigue in MG needs to be treated independently of muscle fatigability.

## Funding Information

This study was supported by the Deutsche Forschungsgemeinschaft (Grant/Award Number: Exc 257).

## Conflicts of Interest

This study was supported by the Deutsche Forschungsgemeinschaft (DFG Exc 257 to Andreas Meisel). Andreas Meisel and Siegfried Kohler have received speaker's honoraria from Novartis and Biomarin and a research grant from Novartis. Sarah Hoffmann, Johanna, Ramm, Ulrike Grittner, and Jana Siedler report no disclosures.
